# *De Novo* Cholesterol Biosynthesis and Its Trafficking in LAMP-1-Positive Vesicles Are Involved in Replication and Spread of Marek’s Disease Virus

**DOI:** 10.1128/JVI.01001-20

**Published:** 2020-11-23

**Authors:** Nitish Boodhoo, Nitin Kamble, Shahriar Behboudi

**Affiliations:** a The Pirbright Institute, Pirbright, Woking, United Kingdom; b Faculty of Health and Medical Sciences, School of Veterinary Medicine, University of Surrey, Guildford, Surrey, United Kingdom; Northwestern University

**Keywords:** Marek’s disease virus

## Abstract

Marek’s disease virus (MDV) transforms CD4^+^ T cells and causes a deadly neoplastic disease that is associated with metabolic dysregulation leading to atherosclerosis in chickens. While MDV-infected chickens have normal serum concentrations of cholesterol, their aortic tissues were found to have elevated concentrations of free and esterified cholesterol. Here, we demonstrate that infection of chicken embryonated fibroblasts (CEFs) with highly pathogenic MDV-RB1B increases the cellular cholesterol content and upregulates the genes involved in cholesterol synthesis and cellular cholesterol homeostasis using comprehensive two-dimensional gas chromatography-mass spectrometry and real-time PCR (RT-PCR), respectively. Using small pharmacological inhibitors and gene silencing, we established an association between MDV-RB1B replication and mevalonic acid, sterol, and cholesterol biosynthesis and trafficking/redistribution. We propose that MDV trafficking is mediated by lysosome-associated membrane protein 1 (LAMP-1)-positive vesicles based on short hairpin RNA (shRNA) gene silencing and the colocalization of LAMP-1, glycoprotein B (gB) of MDV, and cholesterol (filipin III) fluorescence signal intensity peaks. In conclusion, our results demonstrate that MDV hijacks cellular cholesterol biosynthesis and cholesterol trafficking to facilitate cell-to-cell spread in a LAMP-1-dependent mechanism.

**IMPORTANCE** MDV disrupts lipid metabolism and causes atherosclerosis in MDV-infected chickens; however, the role of cholesterol metabolism in the replication and spread of MDV is unknown. MDV-infected cells do not produce infectious cell-free virus *in vitro*, raising the question about the mechanism involved in the cell-to-cell spread of MDV. In this report, we provide evidence that MDV replication depends on *de novo* cholesterol biosynthesis and uptake. Interruption of cholesterol trafficking within multivesicular bodies (MVBs) by chemical inhibitors or gene silencing reduced MDV titers and cell-to-cell spread. Finally, we demonstrated that MDV gB colocalizes with cholesterol and LAMP-1, suggesting that viral protein trafficking is mediated by LAMP-1-positive vesicles in association with cholesterol. These results provide new insights into the cholesterol dependence of MDV replication.

## Abstract (precis)

MDV disrupts lipid metabolism and causes atherosclerosis in MDV-infected chickens; however, the role of cholesterol metabolism in the replication and spread of MDV is unknown. MDV-infected cells do not produce infectious cell-free virus *in vitro*, raising the question about the mechanism involved in the cell-to-cell spread of MDV. In this report, we provide evidence that MDV replication depends on *de novo* cholesterol biosynthesis and uptake. Interruption of cholesterol trafficking within multivesicular bodies (MVBs) by chemical inhibitors or gene silencing reduced MDV titers and cell-to-cell spread. Finally, we demonstrated that MDV gB colocalizes with cholesterol and LAMP-1, suggesting that viral protein trafficking is mediated by LAMP-1-positive vesicles in association with cholesterol. These results provide new insights into the cholesterol dependence of MDV replication.

## INTRODUCTION

Gallid herpesvirus 2 (GaHV-2), or Marek’s disease virus (MDV), is a highly cell-associated avian alphaherpesvirus that causes deadly lymphoma and immunosuppression in chickens. Cell-free virus particles are produced only by feather follicle epithelial cells, and they are shed in association with skin debris into the environment, which can infect chickens via their respiratory tract. Otherwise, infectious cell-free particles are not produced by any other cell types *in vitro* or *in vivo*. MDV can infect many cell types; however, latency is mainly established in CD4^+^ T cells, which are transformed into lymphoma cells (1–4). We have recently shown that MDV infection modulates cell metabolism and specifically enhances fatty acid synthesis and the formation of lipid droplets (5, 6). It has been suggested that the alteration of lipid metabolism by MDV causes atherosclerosis in infected chickens (7, 8). The influence of cholesterol synthesis on the pathogenesis of Marek’s disease was confirmed in series of experiments showing that the use of a 3-hydroxy-3-methylglutaryl-coenzyme A reductase (HMG-CoA) inhibitor reduces atherosclerotic plaque areas and mortality rates in MDV-infected chickens (9). This study examines the mechanism involved in these *in vivo* observations.

Cholesterol synthesis occurs in the cytosol and endoplasmic reticulum (ER), and the synthesized cholesterol is then rapidly transported via multivesicular bodies (MVBs) to other organelles, including the plasma membrane. As part of *de novo* cholesterol biosynthesis, HMG-CoA catalyzes the conversion of HMG-CoA to mevalonic acid. Thereafter, squalene epoxidase (SqE), in a rate-limiting step for sterol biosynthesis, catalyzes the first oxygenation step in the biosynthesis of lanosterol (10). The final stage involves the conversion of lanosterol to cholesterol, which is distributed via lysosome-associated membrane protein 1 (LAMP-1)-positive vesicles between intracellular compartments, including cell membranes (11–13). The majority of LAMP-1 is directly transported to lysosomes via the *trans*-Golgi network (TGN), while some LAMP-1 is initially transported out of the cell membrane, and it is then internalized and delivered to lysosomes (14). It has been suggested that herpesvirus particles are transported to the extracellular space by the fusion of the plasma membrane with vesicle-containing virus particles (15). MVBs are a suitable microenvironment for herpes simplex virus 1 (HSV-1) replication, and glycoprotein B (gB) of HSV-1 is colocalized with the MVB marker LAMP-1 (16). Interestingly, the final envelopment of varicella-zoster virus, a highly cell-associated alphaherpesvirus similar to MDV, in the cytoplasm occurs at TGN-derived vesicles (17, 18).

Very little information is available relating to the mechanism involved in the transportation of MDV particles and the role of cholesterol trafficking in MDV-infected cells. This study examines the influence of MDV infection on cholesterol metabolism in chicken embryonated fibroblasts (CEFs) and demonstrates that MDV replication and spread are dependent on cholesterol synthesis and uptake. Intriguingly, a key relationship between *de novo* cholesterol biosynthesis and MDV cell-to-cell spread was established. Interruption of trafficking from MVBs by chemical inhibitors or gene silencing reduced MDV titers and cell-to-cell spread. Finally, we demonstrated that MDV gB was colocalized with cholesterol and LAMP-1, suggesting that viral protein trafficking is mediated by LAMP-1-positive vesicles in association with cholesterol.

## RESULTS

### Induction of *de novo* cholesterol biosynthesis in MDV-infected CEFs.

**FIG 1:**
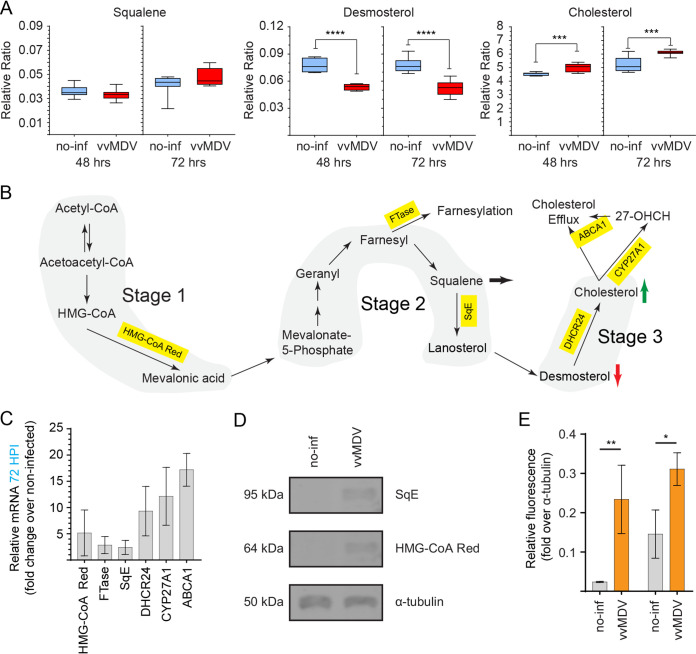
Induction of *de novo* cholesterol synthesis in MDV-infected CEFs. Shown is the alteration of cholesterol and intermediates in MDV-infected CEFs. Data from metabolomics analysis of the relative ratios (ratio of the area to the internal standard) of cholesterol metabolites from mock (noninfected [no-inf])- and MDV-infected (RB1B) CEFs at 48 hpi and 72 hpi are shown. (A) Box-and-whisker plots showing minimum and maximum relative levels of the indicated metabolites identified by metabolome analysis using GC/GC-MS in noninfected or MDV-infected CEFs at 48 and 72 hpi. vvMDV, very virulent MDV. (B) Schematic of cholesterol biosynthesis pathways with a summary of major metabolites outlining the preferential induction of *de novo* cholesterol biosynthesis. Arrows indicate changes in the respective metabolites to demonstrate flux through pathways at 72 h postinfection. (C) Fold changes in gene (HMG-CoA, 3-hydroxy-3-methyl-glutaryl-CoA reductase; SqE, squalene epoxidase; FTase, farnesyl transferase; DHCR24, 24-dehydrocholesterol reductase; ABCA1, ATP-binding cassette transporter 1) expression based on RT-PCR in CEFs infected with MDV over noninfected cells at 72 hpi. (D and E) Relative expression levels of HMG-CoA and SqE proteins in noninfected and MDV-infected CEFs at 72 hpi (D) presented as relative fluorescence intensities over α-tubulin (E). Nonparametric Wilcoxon tests (Mann-Whitney) were used to assess normal distribution and test significance, with the results shown as means ± SD. *** (*P* = 0.0005) and **** (*P* = 0.0001) indicate a statistically significant difference compared to the control. The experiment was performed in biological triplicates with six technical replicates per biological replicate.

The relative levels of three metabolites (squalene, desmosterol, and cholesterol) involved in cholesterol biosynthesis were determined in cell lysates with a total of 6 technical replicates per biological replicate obtained from mock- and MDV-infected primary CEFs at 48 and 72 h postinfection (hpi) using comprehensive two-dimensional gas chromatography-mass spectrometry (GC/GC-MS). The results demonstrated that the levels of desmosterol were lower in MDV-infected cells than in mock-infected cells at both 48 and 72 hpi. In contrast, the cholesterol level was significantly higher in MDV-infected cells than in mock-infected cells at both 48 and 72 hpi (Fig. 1A), suggesting that higher cellular cholesterol contents may have reduced cholesterol synthesis at 48 and 72 hpi. The metabolites and the key enzymes involved in the cholesterol biosynthesis pathway are shown in Fig. 1B. HMG-CoA, farnesyltransferase (FTase), SqE, and 24-dehydrocholesterol reductase (DHCR24) are involved in cholesterol biosynthesis, while CYP27A1 is a gene encoding a cytochrome P450 oxidase that is involved in the degradation of cholesterol. On the other hand, the ATP-binding cassette transporter 1 (ABCA1) gene, expressing the cholesterol efflux regulatory protein, is involved in cellular cholesterol homeostasis (19). Gene expression analyses of the key enzymes involved in cholesterol biosynthesis by real-time PCR (RT-PCR) demonstrate that HMG-CoA, FTase, SqE, DHCR24, CYP27A1, and ABCA1 were upregulated in MDV-infected CEFs at 72 hpi. The upregulation of HMG-CoA and SqE proteins in MDV-infected CEFs was confirmed using a Western blot assay (Fig. 1D and E). The results indicate that cholesterol biosynthesis is upregulated in MDV-infected cells.

**FIG 2:**
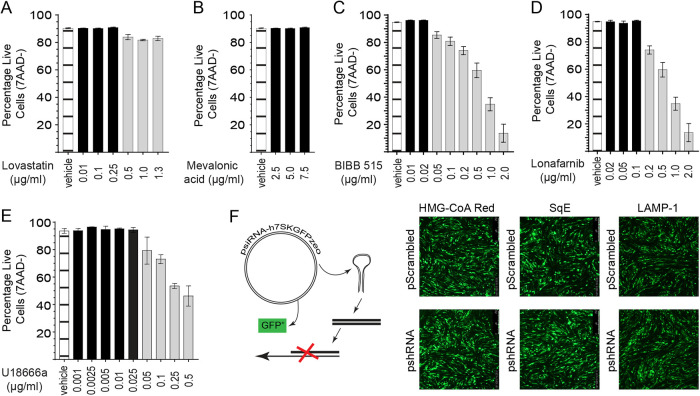
Titration of pharmacological inhibitors and shRNAs targeting rate-limiting enzymes of cholesterol biosynthesis pathways. (A to E) Analysis by MACSQuant demonstrating percentages of live CEFs (7-AAD^−^) after 72 h of treatment with various concentrations of small pharmacological inhibitors, including lovastatin (inhibitor of HMG-CoA) (A), mevalonic acid (B), BIBB 515 (inhibitor of SqE) (C), lonafarnib (inhibitor of FTase) (D), and U18666A (inhibitor of MVB) (E). Bar graphs with single bars in black represent concentrations of the inhibitors that did not induce cell death compared to vehicle treatment. (F) Transfection efficiency of the psiRNA-h7SKGFPzeo plasmid expressing pshRNA or pScrambled as identified by GFP fluorescence at 24 h posttransfection and targeting HMG-CoA, SqE, and LAMP-1 in CEFs. All experiments were performed in triplicates, and data are representative of results from 4 independent experiments performed in parallel with virus infection studies. Vehicle indicates the lovastatin, BIBB 515, lonafarnib, and U18666A carrier (DMSO) (maximum of a 1:500 ratio of culture medium) as well as the mevalonic acid carrier (RPMI 1640).

Going forward, we present a comprehensive analysis of small pharmacological inhibitors and short hairpin RNAs (shRNAs) targeting key enzymes within the cholesterol biosynthesis pathway to elucidate the relative contribution of *de novo* cholesterol to MDV replication and spread. All inhibitors were titrated to determine nontoxic concentrations of lovastatin (Fig. 2A), mevalonic acid (Fig. 2B), BIBB 515 (Fig. 2C), lonafarnib (Fig. 2D), and U18666A (Fig. 2E) based on the confluence of the cells using light microscopy and the presence of live-cell (7-aminoactinomycin D negative [7-AAD^−^]) staining using flow cytometry. In addition, shRNAs targeting the knockdown (green fluorescent protein-positive [GFP^+^] cells) of the respective enzymes HMG-CoA, SqE, and LAMP-1 were used to validate the efficiency of the small pharmacological inhibitors utilized (Fig. 2F).

### MDV infectivity is dependent on *de novo* mevalonic acid biosynthesis.

**FIG 3:**
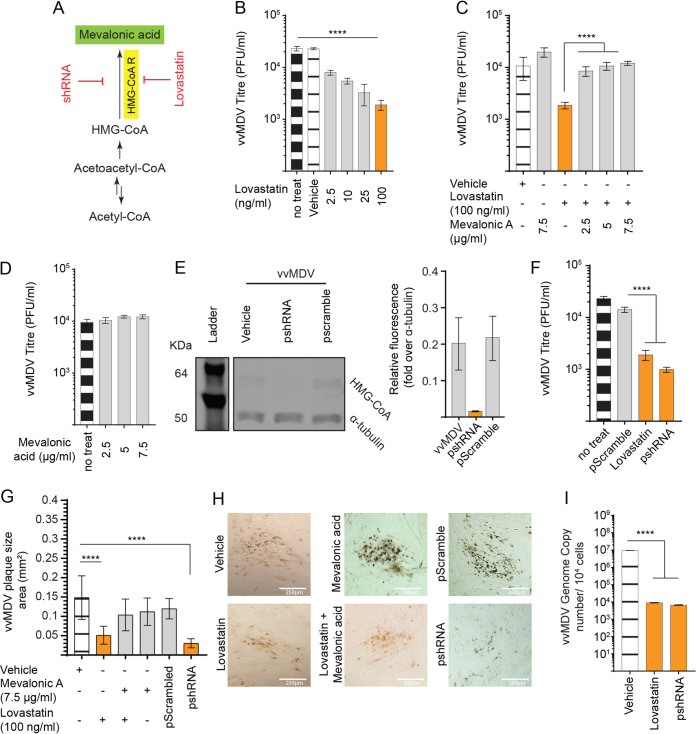
The mevalonate pathway is required for MDV infection of CEFs. (A) Schematic of the mevalonate biosynthesis pathway highlighting the relevantpharmacological inhibitor (red) and the respective enzyme (yellow box) as well as the metabolite (green box) studied based on metabolites identified by metabolome analysis using GC/GC-MS as the primary committed step to *de novo* cholesterol biosynthesis. (B to D) Analysis of MDV titers (PFU per milliliter) in CEFs in the presence of lovastatin (2.5, 10, 25, and 100 ng/ml) alone (B) or in combination with mevalonic acid (2.5, 5, and 7.5 μg/ml) (C) or only mevalonic acid (2.5, 5, and 7.5 μg/ml) (D) at 72 hpi. (E) Relative expression levels of HMG-CoA protein (∼64 kDa) determined by Western blotting in MDV-infected CEFs transfected with pshRNA or pScrambled at 72 hpi, presented as relative fluorescence intensity over α-tubulin. (F) MDV titers in CEFs transfected with pshRNA or pScrambled RNA at 72 hpi. (G) Analysis of MDV spread based on plaque size areas (square millimeters) in the presence of lovastatin alone (100 ng/ml) or in combination with mevalonic acid (7.5 μg/ml), mevalonic acid only (7.5 μg/ml), or pshRNA knockdown of HMG-CoA. (H) Representative pictures of MDV plaques taken at a ×10 magnification using an inverted light microscope. (I) MDV genome copy numbers per 10^4^ cells (Meq gene with the reference ovotransferrin gene) were determined using qPCR on DNA samples extracted from CEFs treated with lovastatin (100 ng/ml) or pshRNA at 72 hpi. *** (*P* = 0.0002) and **** (*P* < 0.0001) indicate a statistically significant difference compared to the control (no treat) or vehicle. Vehicle indicates the lovastatin carrier (DMSO) (maximum of a 1:500 ratio of culture medium) and mevalonic acid carrier (RPMI 1640). All viral titer experiments were performed in 6 replicates, and data are representative of results from 3 independent experiments.

Mevalonic acid is a key metabolite in the synthesis of cholesterol, and HMG-CoA, a rate-controlling enzyme of the mevalonate pathway, is an essential enzyme within the first stage of cholesterol biosynthesis (19). Here, we examined the role of HMG-CoA in the replication of MDV using a small pharmacological inhibitor, lovastatin, and gene silencing by shRNA (Fig. 3A). To this end, we initially determined nontoxic concentrations of lovastatin and mevalonic acid based on the confluence of the cells using light microscopy and 7-AAD staining using flow cytometry. MDV titers were determined in MDV-infected CEFs in the presence of different concentrations of lovastatin. The results demonstrated that nontoxic concentrations of lovastatin (2.5, 10, 25, and 100 ng/ml) reduced MDV titers in a dose-dependent manner (Fig. 3B). Treatments of the cells with 2.5, 10, 25, and 100 ng/ml lovastatin reduced MDV titers in a dose-dependent manner. Exogenous mevalonic acid (2.5 to 7 μg/ml) rescued the inhibitory effects of lovastatin (100 ng/ml) on MDV titers (Fig. 3C), while it did not have any effect on MDV titers in the absence of lovastatin (Fig. 3D). RNA silencing reduced HMG-CoA protein levels to nondetectable levels (Fig. 3E) and reduced MDV titers (Fig. 3F), MDV plaque sizes (Fig. 3G and H), and MDV copy numbers (Fig. 3I).

### *De novo* lanosterol synthesis supports MDV replication in CEFs.

**FIG 4:**
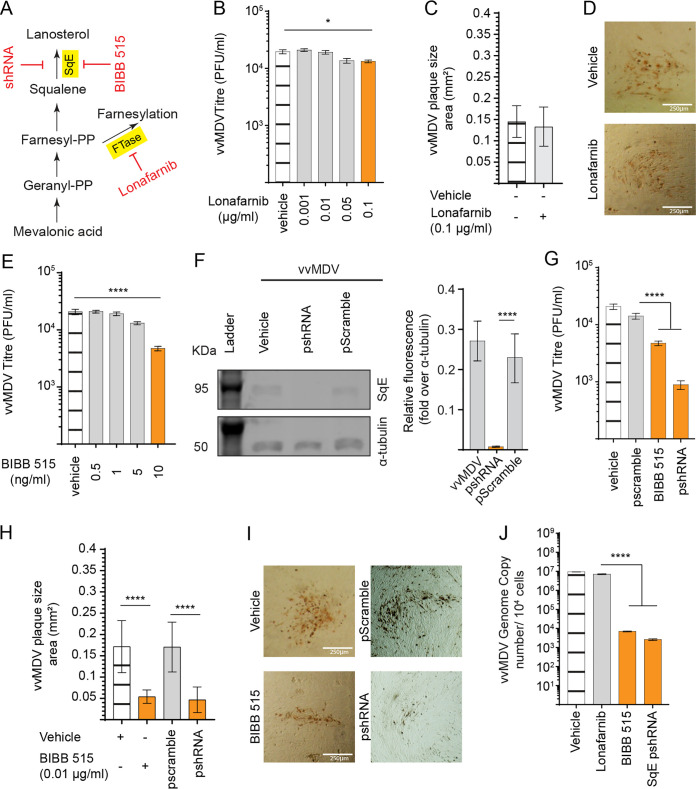
The lanosterol synthesis pathway is required for very virulent (vv) MDV infection of CEFs. (A) Schematic of the lanosterol biosynthesis pathway highlighting the relevant pharmacological inhibitor (red) and the respective enzyme (yellow box) studied based on metabolites (including geranyl pyrophosphate [Geranyl-PP] and farnesyl pyrophosphate [Farnesyl-PP]) identified by metabolome analysis using GC/GC-MS. (B and C) Analysis of MDV titers (PFU per milliliter) (B) and plaque size areas (square millimeters) (C) in the presence of different nontoxic concentrations of the chemical inhibitor of FTase, lonafarnib, or the vehicle at 72 hpi. (D) Representative pictures of MDV plaques taken at a ×10 magnification using an inverted light microscope at 72 hpi. (E to G) Analysis of MDV titers (PFU per milliliter), (E) relative expression levels of SqE protein (∼95 kDa) determined by Western blotting in MDV-infected CEFs transfected with pshRNA or pScrambled at 72 hpi presented as relative fluorescence intensities over α-tubulin (F), and plaque size areas (square millimeters) (G) in the presence of different nontoxic concentrations of the chemical inhibitor of SqE, BIBB 515, or in SqE gene knockdown CEFs using shRNA. (H) Representative pictures of MDV plaques in CEFs treated with BIBB 515 or SqE knockout CEFs taken at a ×10 magnification using an inverted light microscope at 72 hpi. (I) MDV genome copy numbers per 10^4^ cells (Meq gene with the reference ovotransferrin gene) were determined using qPCR in MDV-infected CEFs treated with lonafarnib (0.1 μg/ml), BIBB 515 (10 ng/ml), or psiRNA. * (*P* = 0.01) and **** (*P* < 0.0001) indicate a statistically significant difference compared to the control (no treat). Vehicle indicates the lonafarnib carrier (DMSO) (maximum of a 1:500 ratio of culture medium) and the BIBB 515 carrier (DMSO) (maximum of a 1:500 ratio of culture medium). All viral titer experiments were performed in 6 replicates, and data are representative of results from 3 independent experiments.

The second stage in cholesterol biosynthesis is the lanosterol pathway (Fig. 1B), which preferentially utilizes mevalonic acid to generate lanosterol. In this stage, squalene is converted to lanosterol by SqE (Fig. 4A), while FTase, a cellular enzyme essential for the prenylation of cellular factors, is involved in a posttranslational protein modification process. To examine the role of SqE and FTase in MDV replication, we utilized both small pharmacological inhibitors and gene silencing using the shRNA system. Nontoxic concentrations of lonafarnib, an inhibitor of FTase, did not alter MDV titers (Fig. 4B) or plaque sizes (Fig. 4C and D). In contrast, nontoxic concentrations of BIBB 515, an inhibitor of SqE, significantly reduced MDV titers (Fig. 4E). The silencing of SqE by shRNA reduced protein levels (Fig. 4F), MDV titers (Fig. 4G), plaque sizes (Fig. 4H and I), and MDV copy numbers (Fig. 4J). Taken together, the results demonstrated that *de novo* synthesis of cholesterol is important for MDV replication and spread.

### Exogenous cholesterol is also required for MDV replication in CEFs.

**FIG 5:**
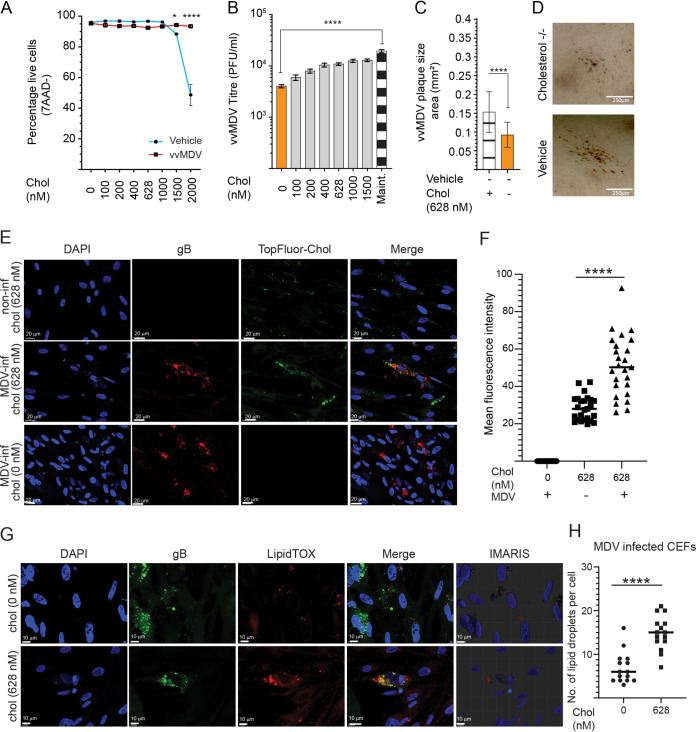
Cholesterol is essential for MDV replication. Analysis was performed to determine the contribution of exogenous cholesterol to MDV replication. (A) Mock- and MDV-infected CEF viability based on 7-AAD^−^ cells as analyzed by fluorescence-activated cell sorting (FACS) at 72 hpi in the presence of various concentrations of cholesterol. (B) Analysis of MDV titers (PFU per milliliter) in CEFs cultured in the presence of exogenous cholesterol (0, 100, 200, 400, 628, 1,000, and 1,500, and complete nM). (C) Analysis of MDV plaque size areas (square millimeters) in the presence/absence of cholesterol (628 nM). (D) Representative pictures, taken at a ×10 magnification using an inverted light microscope, demonstrating differential plaque sizes for the various treatments performed at 72 hpi. (E) Representative pictures showing the uptake of TopFluor cholesterol (green) in mock- and RB1B-infected (red) CEF cells. (F) MFI per cell of TopFluor cholesterol in mock-infected versus RB1B-infected CEF cells in cholesterol-containing (628 nM) and knockout media. (G) Representative pictures demonstrating the maximum projection of z-stacks for each channel showing lipid droplets (red) in mock- and RB1B-infected (green) individual CEF cells. (H) IMARIS panel showing three-dimensional representative pictures of lipid droplets surrounding 4′,6-diamidino-2-phenylindole (DAPI) (blue)-stained nuclei, which were used to calculate lipid droplets per cell in mock- and RB1B-infected CEFs cells. *** (*P* = 0.0002) and **** (*P* < 0.0001) indicate a statistically significant difference compared to the control (no treat). Vehicle indicates the cholesterol carrier (MilliQ water). Maintenance medium (Maint.) was used for optimal viral replication. All viral titer experiments were performed in 6 replicates from 3 independent experiments.

To determine the importance of exogenous cholesterol in MDV replication, we examined the cell viability of mock- or MDV-infected CEFs in cell culture medium with different concentrations of cholesterol (ranging from 0 to 2,000 nM). High levels of cholesterol (1,500 and 2,000 nM) were toxic to mock-infected CEFs, while these levels of cholesterol were nontoxic to MDV-infected CEFs as determined based on cell viability using 7-AAD staining (Fig. 5A), suggesting that MDV infection alters the susceptibility of the cells to exogenous cholesterol. To further interrogate the role of exogenous cholesterol in MDV replication, we analyzed MDV titers in MDV-infected CEFs that were cultured in medium containing different concentrations of cholesterol. Lower MDV titers (Fig. 5B) and smaller plaque sizes (Fig. 5C) were observed in MDV-infected CEFs that were cultured in cell culture medium lacking exogenous cholesterol. Furthermore, the result demonstrated that addback of cholesterol increased MDV titers (Fig. 5B). Furthermore, the fate of exogenous cholesterol added to infection medium was determined by adding fluorescent TopFluor cholesterol to culture media of RB1B- and mock-infected CEF cells. Both RB1B- and mock-infected CEF cells showed uptake of TopFluor cholesterol from the surrounding medium (Fig. 5E). Furthermore, quantification of the mean fluorescence intensity (MFI) showed significantly higher intensity and uptake of TopFluor cholesterol preferentially in CEF cells infected with RB1B (average MFI of 50.9) than in mock-infected cells (average MFI of 28.4) (Fig. 5F). Also, RB1B-infected CEF cells cultured in the presence of exogenous cholesterol showed significantly higher accumulation of lipid droplets (average of 15 droplets per cell) than in RB1B-infected CEF cells cultured without exogenous cholesterol (average of 7 droplets per cell) (Fig. 5G and H). Taken together, our data demonstrate that exogenous and *de novo*-synthesized cholesterol are involved in MDV replication and spread.

### Modulation of cholesterol trafficking impairs MDV replication and spread.

**FIG 6:**
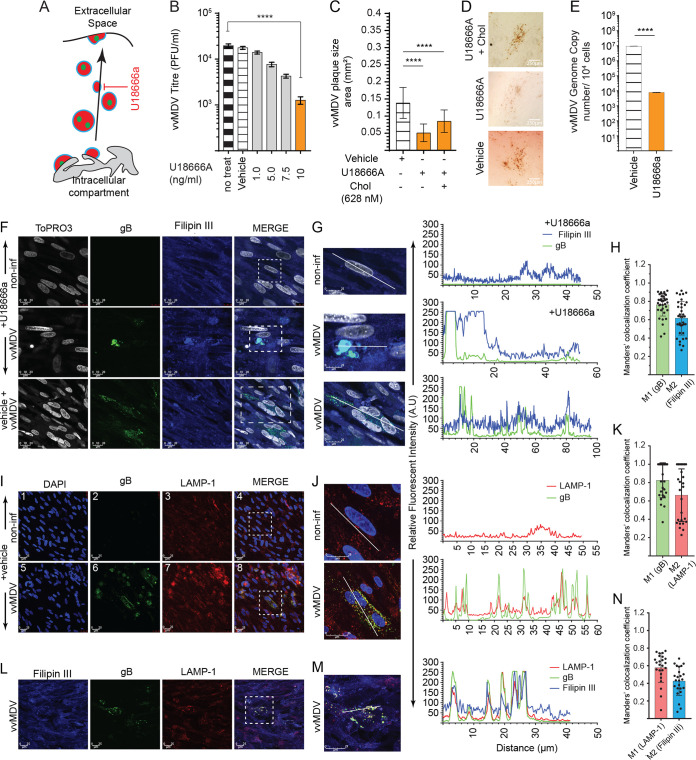
Inhibition of intracellular vesicle transport inhibits MDV spread. (A) Targeted interference of cholesterol trafficking in MVBs by the U18666A smallpharmacological inhibitor and shRNA. (B and C) Analysis of MDV titers (PFU per milliliter) (B) and plaque size areas (square millimeters) (C) in the presence of various nontoxic concentrations of U18666A (0.75, 1.0, 5.0, 7.5, and 10 ng/ml) alone or in combination with cholesterol (628 nM) at 72 hpi. (D) Representative pictures, taken at a ×10 magnification using an inverted light microscope, demonstrating differential plaque sizes from the treatment groups at 72 hpi. (F, G, I, J, L, and M) Confocal microscopy imaging with maximum projections of z-stacks for each channel demonstrating the cytoplasmic distribution in MDV-RB1B (gB) (green)- and mock-infected CEFs treated with either U18666A (10 ng/ml), the vehicle, or cholesterol (filipin III) (blue) and lysosome-associated membrane protein 1 (LAMP-1) (red). (H, K, and N) Validation of colocalization efficiencies by Manders’ colocalization coefficient as analyzed using ImageJ 72 h after infection with MDV. Colocalizations between gB (M1) and filipin III (M2) (H), gB (M1) and LAMP-1 (M2) (K), and LAMP-1 (M1) and filipin III (M2) (N) represent the overlap of M1 as the denominator and vice versa (*n *=* *30). Maximum projections of all combined channels (merge) were analyzed using LAS AF Lite software to determine the relative fluorescence intensity (arbitrary units [A.U]) profiles from selected (white dashed boxes) noninfected and MDV-infected CEFs treated with either U18666A or the vehicle only for gB (green) and filipin III (blue) (G), gB (green) and LAMP-1 (red) (J), and gB (green), filipin III (blue), and LAMP-1 (red) (M) along the white line path (micrometers) indicated in the corresponding ROIs (merge) demonstrating overlapping signals. *** (*P* = 0.0002) and **** (*P* < 0.0001) indicate a statistically significant difference compared to the control (no treat) or vehicle. Vehicle indicates the U18666A carrier (DMSO) (maximum of a 1:500 ratio of culture medium). All viral titer experiments were performed in 6 replicates, and the data are representative of results from 3 independent experiments.

U18666A, a chemical inhibitor that inhibits late endosomes (MVBs) and cholesterol transport (Fig. 6A), was utilized to study the role of cholesterol trafficking in MDV replication and spread. The results demonstrate that inhibition of cholesterol trafficking by nontoxic concentrations of U18666A significantly reduced MDV titers (Fig. 6B) and plaque sizes (Fig. 6C). Exogenous cholesterol (628 nM) did not rescue the inhibitory effects of U18666A (10 ng/ml) on MDV spread (Fig. 6C and D). Furthermore, a significant reduction in MDV genome copy numbers was observed in U18666A-treated MDV-infected CEFs compared to vehicle-treated cells (Fig. 6E).

The colocalization of filipin III, a fluorescent probe for cholesterol, and MDV (gB of MDV) was analyzed using confocal immunofluorescence microscopy. Vehicle-treated MDV-infected CEFs showed a diffuse distribution of cholesterol (Fig. 6F and G), and the peak relative fluorescence intensity for filipin III was associated with gB of MDV (Fig. 6G). Manders’ coefficient for filipin III and gB of MDV confirms the colocalization of cholesterol and the MDV viral protein (Fig. 6H). The results also demonstrated that U18666A disrupted both cholesterol and gB distributions in MDV-infected CEFs (Fig. 6G).

The relative expression levels and colocalization of LAMP-1, a lysosomal marker, and gB of MDV were analyzed in MDV-infected CEFs using confocal microscopy. The results demonstrate that MDV-infected CEFs have higher expression levels of LAMP-1 as identified by the higher fluorescence intensity in MDV-infected cells than in mock-infected CEFs. Moreover, LAMP-1 and gB of MDV are colocalized in infected cells (Fig. 6I and J). Manders’ coefficient for LAMP-1 and gB of MDV confirms these observations (Fig. 6K), suggesting the involvement of the MVB pathway in MDV replication. The results also demonstrate a relationship between cholesterol (filipin III), LAMP-1, and gB in MDV-infected CEFs (Fig. 6H and K to N).

### LAMP-1 is essential for efficient replication and spread of MDV.

**FIG 7:**
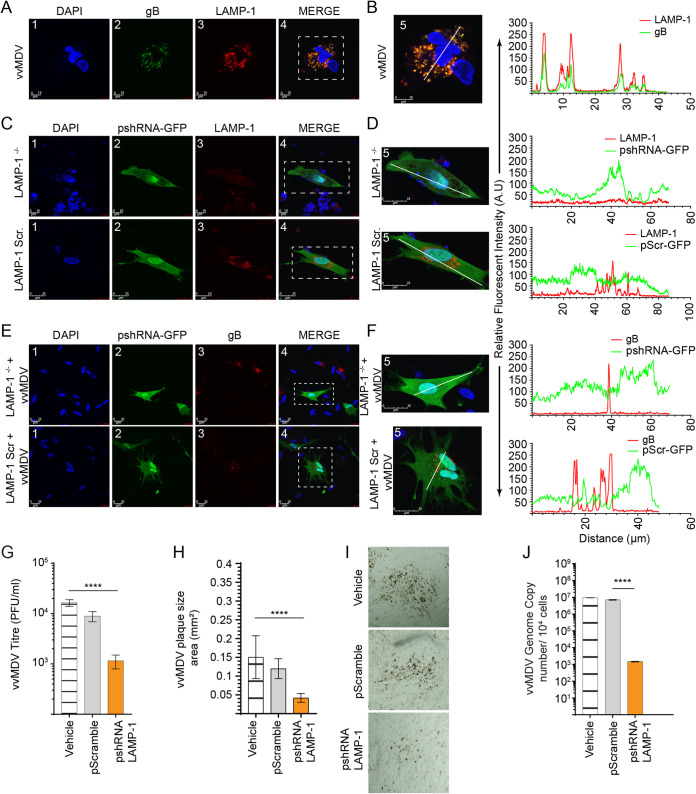
LAMP-1 facilitates MDV cell-to-cell spread. Targeted avian LAMP-1 knockdown using an shRNA expressing the psiRNA-h7SKGFPZeo plasmid (GFP^+^)demonstrates the lack of MDV spread. (A, C, and E) Representative visualization, at 72 hpi, by confocal microscopy imaging with maximum projections of z-stacks for each channel demonstrating the cytoplasmic distribution of LAMP-1 in MDV-RB1B (gB [green] and LAMP-1 [red]) (A) or the pshRNA-expressing plasmid targeting silencing (LAMP-1^−/−^) or scrambled (Scr) in noninfected (pshRNA [green] and LAMP-1 [red]) (C) and MDV-infected (pshRNA [green] and gB [red]) (E) CEFs. (B, D, and F) Maximum projections of all combined channels (merge) were analyzed using LAS AF Lite software to determine the relative fluorescence intensity (arbitrary units [A.U]) profiles from selected (white dashed boxes) noninfected and MDV-infected CEFs for MDV-RB1B gB (green) and LAMP-1 (red) (B), a pshRNA-expressing plasmid (LAMP-1^−/−^ or Scr [green]) and LAMP-1 (red) in noninfected CEFs (D), and a pshRNA-expressing plasmid (LAMP-1^−/−^ or Scr [green]) in MDV-RB1B gB (red)-infected CEFs (F) along the white line path (micrometers) indicated in the corresponding ROIs (merge) demonstrating overlapping signals. (G and H) Analysis, at 72 hpi, of MDV titers (PFU per milliliter) (G) and plaque size areas (square millimeters) (H) in CEFs expressing shRNA for targeted LAMP-1 silencing (pshRNA) or scrambled (pScramble). (I) Representative pictures, taken at a ×10 magnification using an inverted light microscope, demonstrating differential plaque sizes for the various treatments performed at 72 hpi. (J) MDV genome copy numbers per 10^4^ cells (MEQ gene with the reference ovotransferrin gene) were determined using qPCR in CEFs expressing shRNA for targeted LAMP-1 silencing (pshRNA) or scrambled (pScramble). **** (*P* < 0.0001) indicates a statistically significant difference compared to the vehicle or pScramble. All viral titer experiments were performed in 6 replicates, and the data are representative of results from 3 independent experiments.

To corroborate the importance of LAMP-1 in MDV infection, we initially verified the shRNA silencing of LAMP-1 and showed a downregulation of LAMP-1 protein in CEFs using confocal microscopy (Fig. 7A and B). Interestingly, shRNA silencing of LAMP-1 in MDV-infected CEFs reduced the expression of gB in MDV-infected CEFs, while transfection with pScramble shRNA had no effect on gB expression in these cells (Fig. 7C and D). The results also demonstrate that shRNA silencing of LAMP-1 significantly reduced MDV titers (Fig. 7E), plaque sizes (Fig. 7F and G), and viral copy numbers (Fig. 7H). Taken together, the results demonstrate that LAMP-1 is required for the efficient replication and spread of MDV, which is consistent with the involvement of the MVB pathway in MDV replication.

## DISCUSSION

The salient findings of this study are that (i) MDV infection increases the cholesterol content of infected cells and activates *de novo* cholesterol synthesis; (ii) exogenous and *de novo* cholesterol biosynthesis are required for efficient MDV replication and spread; (iii) MDV induces the upregulation of LAMP-1, involved in cholesterol trafficking, within multivesicular bodies (MVBs); and (iv) glycoprotein B of MDV, an essential viral protein for replication and egress, is associated with cholesterol and LAMP-1 within MVBs. Interestingly, gene silencing of LAMP-1 reduced both MDV replication and spread, highlighting the importance of LAMP-1 as a protein for the transportation of gB. The results suggest that MDV hijacks cellular cholesterol biosynthesis and cholesterol trafficking to facilitate cell-to-cell spread in a LAMP-1-dependent mechanism. The results from this study could be used to design control strategies to use chemical or physiological inhibitors targeting the cholesterol pathway to reduce virus replication and shedding. Alternatively, novel vaccines such as herpesvirus of turkeys (HVT) vaccines expressing shRNA for silencing the cholesterol pathway could be developed to reduce MDV replication *in vivo*.

MDV is a highly cell-associated alphaherpesvirus that infects chickens and causes deadly lymphoma and immunosuppression (1, 2, 20). Although MDV infection disturbs lipid metabolism and causes atherosclerosis (7–9), very little is known about the role of MDV-induced cholesterol metabolism in MDV replication and spread. MDV can infect various cell types; however, it transforms only CD4^+^ T cells (1, 21). *In vitro*, MDV infection of lymphocytes occurs only after the activation of B and T cells (22), which can mask a metabolic alteration under MDV infection (2, 23). Therefore, here, we analyzed the role of cholesterol in MDV infection in CEFs, which can be infected with an inoculum without any external activation. CEFs contain various cell populations, including immune cells and fibroblasts, which are used for the propagation of MDV. The exact proportion of immune cells within CEFs is unknown as there are no specific antibodies to detect all different types of chicken immune cells, and many immune cells might be in their progenitor stage and do not express specific markers. Based on our previous observations, there are only very small percentages of major histocompatibility complex (MHC) class II-positive or KULO1-positive cells representing macrophages and CD3^+^ cells representing T cells within CEFs, suggesting that the majority of infected cells within CEFs are not macrophages or T cells. Therefore, it is not possible to determine the impact of immune cell infection within CEFs on the results observed in this study. Inhibition of the cholesterol pathway reduces MDV replication *in vivo*, and thus, we believe that the cholesterol pathway is involved in MDV replication even in the presence of the immune system. However, further research is required to determine the role of the cholesterol pathway in MDV replication in immune cells. At 72 hpi, in our MDV infection culture model, over 80% of CEFs were infected and induced maximum virus infection. At later time points, cell detachment and death occurred, which can affect cell metabolism and mask the effects of MDV infection on cholesterol biosynthesis. Thus, cholesterol biosynthesis was analyzed in the samples at 48 and 72 hpi.

We have recently observed that MDV infection increases the formation of lipid droplets (5), which play an important role in sustaining the cellular cholesterol level by maintaining lipid storage, hydrolysis, and trafficking. The biosynthesis, efflux, and influx of cholesterol regulate the levels of cholesterol in cells and, together with cholesterol distribution and dynamics, regulate cholesterol’s function. Studies have revealed that the depletion of cholesterol can inhibit herpes simplex virus entry by inhibiting cell binding and/or changing the expression levels of receptors on the cell surfaces (24). Moreover, cholesterol and lipid rafts can also be involved in virus replication by influencing the intracellular membrane structures (25). MDV is a highly cell-associated virus, and the role of cholesterol in MDV cell-to-cell spread and replication needs to be further explored. This study demonstrated that genes involved in cholesterol synthesis and trafficking are upregulated and that the levels of cholesterol in MDV-infected cells are increased, suggesting that MDV infection increases cholesterol biosynthesis. Interestingly, MDV-infected cells became resistant to cytotoxicity, which is generally exerted by excessive exogenous cholesterol. Cell apoptosis is triggered by the enrichment of free cholesterol in the ER (26), which plays a major role in protein homeostasis and cholesterol production. It is possible that the usage and trafficking of free cholesterol remove free cholesterol from the ER in MDV-infected cells. Our confocal microscopy data demonstrating the colocalization of cholesterol and gB of MDV within LAMP-1 MVBs support this notion. To our knowledge, this is the first report confirming the crucial role of cholesterol synthesis and trafficking in MDV replication and spread.

LAMP-1 shuttles between lysosomes, endosomes, and the plasma membrane (27) and binds to cholesterol in association with Niemann-Pick disease type C1 (NPC1) and NPC2 proteins that export cholesterol from lysosomes (28). Our results demonstrated that MDV infection enhanced the expression of LAMP-1, a marker for the MVB compartment, reflecting the augmentation and involvement of the MVB pathway in MDV replication. The mechanism involved in the upregulation of LAMP-1 during MDV infection is unknown, and further work is required to establish any possible association between cholesterol synthesis and LAMP-1 expression in MDV-infected cells. Confocal microscopy showed the colocalization of gB with cholesterol and LAMP-1 within MVB membranes. MDV-encoded gB is one of the essential MDV glycoproteins, which is conserved in most herpesviruses, and here, we show that gB may be sorted to the MVB compartment, and confocal microscopy showed that gB was colocalized in part with the MVB membranes, which therefore represent a site of gB accumulation. Herpesvirus glycoproteins play an important role in the assembly and production of infectious particles, and gB of herpesviruses recirculates between the plasma membrane and MVBs (16, 29, 30). RNA interference with LAMP-1 reduced MDV titers and plaque sizes, highlighting the importance of LAMP-1 and MVBs in MDV replication and cell-to-cell spread. It has been suggested that avian LAMP-1 is involved in the degradation of the avian reovirus nonstructural p10 protein, involved in the induction of cell syncytium formation and apoptosis, through interacting with both p10 and the E3 ligase Siah-1 (31, 32). We are currently examining whether LAMP-1 is also involved in the degradation of MDV viral proteins, which are required for the generation of infectious cell-free MDV. Feather follicle epithelial cells are the only cells capable of producing infectious cell-free MDV *in vivo*, and we have recently generated feather follicle epithelial cell lines that will be used to differentially examine the role of LAMP-1 in the degradation of MDV proteins. In conclusion, the results suggest that MDV activates LAMP-1 expression and hijacks cellular cholesterol biosynthesis and cholesterol trafficking to facilitate MDV trafficking in a LAMP-1-dependent mechanism.

## MATERIALS AND METHODS

### Ethics statement.

Ten-day-old mixed-sex specific-pathogen-free (SPF) embryonated chicken eggs, which were purchased from Valo Biomedia GmbH, were used to generate primary CEFs. All embryonated chicken eggs were handled in strict accordance with the guidance and regulations of European and United Kingdom Home Office regulations under project license number 30/3169. The experiments were approved by the ethics committee at The Pirbright Institute.

### CEF culture and virus preparations.

CEFs were generated from mixed-sex SPF Valo eggs (Valo Biomedia GmbH) incubated in a Brinsea Ova-Easy 190 incubator at 37°C until 10 days *in ovo*. CEFs were seeded at a rate of 1.5 × 10^5^ cells/ml in 24-well plates with growth medium (E199 medium supplemented with 10% TBP, 5% fetal calf serum [FCS], 2.8% Milli-Q-filtered water, amphotericin B [0.01%], penicillin [10 U/ml], and streptomycin [10 μg/ml]) and incubated overnight (38.5°C at 5% CO_2_). The next day, 80% confluent monolayers were observed, and growth medium was replaced with maintenance medium (E199 medium supplemented with 10% tryptose phosphate broth (TPB) 2.5% FCS, 3.5% SQ water, amphotericin B [0.01%], penicillin [10 U/ml], and streptomycin [10 μg/ml]).

### Reagents and antibodies. (i) Chemicals.

Lovastatin (stock; 2.5 mg/ml), BIBB 515 (stock; 2.5 mg/ml), lonafarnib (stock; 1 mg/ml) (Cambridge Bioscience, Cambridge, UK), and U18666A (stock; 5 mg/ml) (Sigma-Aldrich/Merck, Dorset, UK) were reconstituted in dimethyl sulfoxide (DMSO). Mevalonic acid lithium salt (stock; 10 mg/ml) and water-soluble cholesterol (stock; 300 mg/ml) (Sigma-Aldrich/Merck, Dorset, UK) were reconstituted in E199 medium. Filipin III was used as part of a kit and reconstituted in cholesterol detection assay buffer prior to use (Abcam, Cambridge, UK). TopFluor cholesterol (Sigma-Aldrich/Merck, Dorset, UK) was reconstituted in ethanol. HCS LipidTOX (Thermo Fisher Scientific, Paisley, UK) was reconstituted in DMSO.

### (ii) Antibodies.

Primary antibodies used were mouse monoclonal antibody (mAb) to chicken α-tubulin, rabbit mAb (IgG) to HMG-CoA (Cambridge Bioscience, Cambridge, UK), and rabbit polyclonal antibody (pAb) to squalene epoxidase (Sigma-Aldrich/Merck, Dorset, UK). The LEP100 hybridoma line (anti-LAMP-1; mouse IgG1 isotype) was obtained through the Developmental Studies Hybridoma Bank (DSHB) (University of Iowa, Iowa City, IA, USA). The HB3 hybridoma line (anti-gB; mouse IgG2b isotype) is an in-house-available hybridoma. Antibodies were purified in-house by a high-performance liquid chromatography (HPLC) method. Secondary antibodies used were goat pAb to rabbit IgG (Abcam, Cambridge, UK), goat anti-mouse IgG1-568 nm, and goat anti-mouse IgG2b-568 nm (Thermo Fisher Scientific, Paisley, UK).

### Cells and MDV infection: metabolomics.

CEFs were either mock infected or infected with the very virulent RB1B strain (100 PFU per 1.5 × 10^5^ cells or a multiplicity of infection [MOI] of 0.0006) in triplicates and harvested at 48 and 72 h postinfection (hpi). The cells were washed and counted; after protein quantification using the Bradford assay (33), the samples were sent for metabolome analysis using GC/GC-MS (Target Discovery Institute, University of Oxford); and data analyses were performed as previously described (34). In brief, the cells were homogenized using a bead beater in methanol-water (1:1), and *t*-butyl methyl ether was then added for phase separation. The organic phase was dried under a vacuum, while methanol was added to the remaining sample and mixed in the bead beater. After incubation at −80°C for 1 h, phase separation occurred after centrifugation, and the liquid layer was collected and dried under a vacuum. Methoxyamine and *N*-methyl-*N*-(trimethylsilyl)trifluoroacetamide (MSFTA) were added to the dried samples and subsequently injected for analysis by GC/GC-MS. The lipid profiles of mock- and MDV-infected cells were analyzed in biological triplicates, with up to six technical replicates per biological replicate. The data were adjusted and normalized based on the protein content. MDV infection did not change the size of the cells as determined using light microscopy.

### Viral plaque analysis. (i) Pretreatment of cells with pharmacological inhibitors.

Lovastatin (2.5, 10, 25, and 100 ng/ml), lonafarnib (1, 10, 50, and 100 ng/ml), BIBB 515 (0.5, 1, 5, and 10 ng/ml), U18666A (1, 5.0, 7.5, and 10 ng/ml), mevalonic acid (2.5, 5, and 7.5 μg/ml), and water-soluble cholesterol (100, 200, 400, 628, 1,000, and 1,500 nM) were added to the cell monolayer 2 h prior to infection with the very virulent strain RB1B (100 PFU per 1.5 × 10^5^ cells), and the cells were incubated (38.5°C with 5% CO_2_) for 72 h.

### (ii) Knockdown of HMG-CoA, SqE, and LAMP-1 expression.

Small short hairpin RNAs (shRNAs), both silencing (pshRNA) and control scrambled (pScrambled) RNA sequences, were designed using siRNA Wizard (InvivoGen, Toulouse, France) (Table 1) and chemically synthesized (Sigma-Aldrich/Merck, Dorset, UK). Construction of the shRNA-harboring plasmid was performed according to the manufacturer’s instructions using the psiRNA-h7SKGFPzeo plasmid. The shRNAs were annealed and ligated into the BbsI-digested psiRNA-h7SK-GFPzeo plasmid vector (catalog no. ksirna4-gz21). The plasmid constructs with shRNAs were transformed into chemically competent GT116 Escherichia coli cells for blue/white screening and zeocin selection and amplification. The positive colonies were propagated, and the Maxiprep method was performed for plasmid preparation. For silencing purposes, both psiRNA-h7SKGFPzeo plasmids expressing pshRNA and pScrambled were transfected using lipofection (Lipofectamine 2000; Thermo Fisher Scientific, Paisley, UK) in CEFs at various DNA concentrations to determine the transfection efficiency and toxicity. The transfection efficiency was observed at 24 h posttransfection for GFP fluorescence using an inverted fluorescence microscope, and wells showing a >80% transfection efficiency were infected with MDV.

**TABLE 1:** shRNA sequences used in the study^*a*^

Gene and shRNA	Direction	Sequence (5′–3′)	*T_m_* (°C)
HMG-CoA reductase			
siRNA#1	F	ACCTCGACCGAATCCACGCTTTCATTTCAAGAGAATGAAAGCGTGGATTCGGTCTT	47.62
	R	CAAAAAGACCGAATCCACGCTTTCATTCTCTTGAAATGAAAGCGTGGATTCGGTCG	
scRNA#1	F	ACCTCGTCCTCCAATCGTGCTATCAATCAAGAGTTGATAGCACGATTGGAGGACTT	
	R	CAAAAAGTCCTCCAATCGTGCTATCAACTCTTGATTGATAGCACGATTGGAGGACG	

siRNA#2	F	ACCTCGGAGCAAGGAGCCGTATTCTTTCAAGAGAAGAATACGGCTCCTTGCTCCTT	52.38
	R	CAAAAAGGAGCAAGGAGCCGTATTCTTCTCTTGAAAGAATACGGCTCCTTGCTCCG	
scRNA#2	F	ACCTCGCAAGTTCGCCGTATGAGTGATCAAGAGTCACTCATACGGCGAACTTGCTT	
	R	CAAAAAGCAAGTTCGCCGTATGAGTGACTCTTGATCACTCATACGGCGAACTTGCG	

siRNA#3	F	ACCTCGGTCAGGATGCTGCTCAGAATTCAAGAGATTCTGAGCAGCATCCTGACCT	52.38
	R	CAAAAAGGTCAGGATGCTGCTCAGAATCTCTTGAATTCTGAGCAGCATCCTGACCG	
scRNA#3	F	ACCTCGCGGAAAGCTGTCGTCTATGATCAAGAGTCATAGACGACAGCTTTCCGCTT	
	R	CAAAAAGCGGAAAGCTGTCGTCTATGACTCTTGATCATAGACGACAGCTTTCCGCG	

LAMP-1			
siRNA#1	F	ACCTCGCATAATTGCTAACCTGACAGTCAAGAGCTGTCAGGTTAGCAATTATGCTT	42.86
	R	CAAAAAGCATAATTGCTAACCTGACAGCTCTTGACTGTCAGGTTAGCAATTATGCG	
scRNA#1	F	ACCTCAGCTCTAAACGGTGACATCATTCAAGAGATGATGTCACCGTTTAGAGCTTT	
	R	CAAAAAAGCTCTAAACGGTGACATCATCTCTTGAATGATGTCACCGTTTAGAGCTG	

siRNA#2	F	ACCTCGGGAAGATATGGTCTCTACTATCAAGAGTAGTAGAGACCATATCTTCCCTT	42.86
	R	CAAAAAGGGAAGATATGGTCTCTACTACTCTTGATAGTAGAGACCATATCTTCCCG	
scRNA#2	F	ACCTCACATGGTGATGACCGTTAGATTCAAGAGATCTAACGGTCATCACCATGTTT	
	R	CAAAAAACATGGTGATGACCGTTAGATCTCTTGAATCTAACGGTCATCACCATGTG	

siRNA#3	F	ACCTCATGGTAAAGTCAGCATAAAGATCAAGAGTCTTTATGCTGACTTTACCATTT	33.33
	R	CAAAAAATGGTAAAGTCAGCATAAAGACTCTTGATCTTTATGCTGACTTTACCATG	
scRNA#3	F	ACCTCGAAGGAGAACGCTAAATATTATCAAGAGTAATATTTAGCGTTCTCCTTCTT	
	R	CAAAAAGAAGGAGAACGCTAAATATTACTCTTGATAATATTTAGCGTTCTCCTTCG	

SQLE			
siRNA#1	F	ACCTCGAAGCCTCTGCGTTTCTCTGTTCAAGAGACAGAGAAACGCAGAGGCTTCTT	52.38
	R	CAAAAAGAAGCCTCTGCGTTTCTCTGTCTCTTGAACAGAGAAACGCAGAGGCTTCG	
scRNA#1	F	ACCTCGTGCTCATCCGCGTCATGTTTTCAAGAGAAACATGACGCGGATGAGCACTT	
	R	CAAAAAGTGCTCATCCGCGTCATGTTTCTCTTGAAAACATGACGCGGATGAGCACG	

siRNA#2	F	ACCTCGAGAGCAAGTCGGAGGTAGAATCAAGAGTTCTACCTCCGACTTGCTCTCTT	52.38
	R	CAAAAAGAGAGCAAGTCGGAGGTAGAACTCTTGATTCTACCTCCGACTTGCTCTCG	
scRNA#2	F	ACCTCGGAGCGATAGCGGGATAGAAATCAAGAGTTTCTATCCCGCTATCGCTCCTT	
	R	CAAAAAGGAGCGATAGCGGGATAGAAACTCTTGATTTCTATCCCGCTATCGCTCCG	

siRNA#3	F	ACCTCGGTGCTGATTGGCCACTTCTTTCAAGAGAAGAAGTGGCCAATCAGCACCTT	52.38
	R	CAAAAAGGTGCTGATTGGCCACTTCTTCTCTTGAAAGAAGTGGCCAATCAGCACCG	
scRNA#3	F	ACCTCGCGGAGTTTCGTCCACTTGTTTCAAGAGAACAAGTGGACGAAACTCCGCTT	
	R	CAAAAAGCGGAGTTTCGTCCACTTGTTCTCTTGAAACAAGTGGACGAAACTCCGCG	

a siRNA, small interfering RNA; scRNA, small cytoplasmic RNA; HMG-CoA reductase, 3-hydroxy-3-methyl-glutaryl-CoA reductase; LAMP-1, lysosome-associated membrane protein 1; SQLE, squalene epoxidase; F, forward; R, reverse; *T_m_*, melting temperature.

### (iii) Viral titer.

MDV-infected cells were titrated onto fresh CEFs. Cells were fixed (1:1 ice-cold acetone-methanol for 5 min) and blocked with blocking buffer (phosphate-buffered saline [PBS] plus 5% FCS) for 1 h at room temperature (RT). Cells were subsequently incubated with anti-gB mAb (HB-3) and then with horseradish peroxidase-conjugated rabbit polyclonal anti-mouse IgG. After the development of the plaques using the 3-amino-9-ethylcarbazole (AEC) substrate, the cells were washed with Super Q water, and viral plaques were counted using a light microscope.

### (iv) Determining nontoxic concentrations of the inhibitors.

To identify nontoxic concentrations of the chemicals, mock-infected and MDV-infected CEFs were exposed to the chemicals or vehicles, and cell morphology and adherence/confluence were monitored by light microscopy at different time points posttreatment. Moreover, CEFs were trypsinized, stained with 7-AAD (BD Bioscience, Oxford, UK), and acquired using a MACSQuant flow cytometer and FlowJo software for analysis of the data. Nontoxic concentrations of the inhibitors and chemicals were selected based on flow cytometry data and confluence.

### qPCR to amplify MDV genes.

DNA samples were isolated from 5 × 10^6^ cells using the DNeasy-96 kit (Qiagen, Manchester, UK), according to the manufacturer’s instructions. A master mix was prepared: primers Meq-FP and Meq-RP (0.4 μM), Meq probes (0.2 μM), *ovo* forward and reverse primers (0.4 μM), *ovo* probe (0.2 μM, 5′ Yakima yellow–3′ 6-carboxytetramethylrhodamine [TAMRA]; Eurogentec), and ABsolute Blue quantitative PCR (qPCR) low-Rox master mix (Thermo Fisher Scientific, Paisley, UK). Standard curves generated for both Meq (10-fold serial dilutions prepared from the plasmid construct with a Meq target) and the *ovo* gene (10-fold serial dilutions prepared from the plasmid construct with an *ovo* target) were used to normalize DNA samples and quantify MDV genomes per 10^4^ cells. All reactions were performed in triplicates to detect both *Meq* and the chicken ovotransferrin (*ovo*) gene on an ABI7500 system (Applied Biosystems) under standard conditions. MDV genomes were normalized and reported as viral genomes per 10^4^ cells.

### Plaque size measurement.

There were approximately 70 to 100 plaques in each well, and many plaques were fused with their adjacent plaques or were located at the edge of the wells, which made measuring their sizes very difficult. Therefore, in previous studies, we standardized our methods and measured the sizes of all the plaques that could be measured correctly and compared them with the results from 20 plaques from each well. The data indicated that the size of 20 plaques from each well represents accurate average sizes of measurable plaques. Therefore, at 72 hpi, the treated CEFs were fixed, and virus plaques were visualized using the AEC substrate (as described above). Viral plaques were imaged (10× lens objective) with an inverted light microscope, and the pictures were processed using Adobe Photoshop software. All viral plaques were measured using the ImageJ software area tool (NIH, USA). The freehand tool was used to measure the defined plaque area (square micrometers) based on anti-gB staining as described previously (5, 6). Data were corrected and exported as plaque size areas (square millimeters).

### Real-time PCR. (i) RNA extraction and cDNA.

Total RNA was extracted from mock- and MDV-infected CEFs using TRIzol (Thermo Fisher Scientific, Paisley, UK) according to the manufacturer’s protocol, and the purified RNA was reverse transcribed to cDNA using a Superscript III first-strand synthesis kit (Thermo Fisher Scientific, Paisley, UK) and oligo(dT) primers according to the manufacturer’s recommended protocol.

### (ii) SYBR green real-time PCR.

Quantitative real-time PCR using SYBR green was performed on cDNA using the LightCycler 480 II system (Roche Diagnostics GmbH, Mannheim, Germany) as previously described (6). Briefly, each reaction involved a preincubation step at 95°C for 5 min, followed by 40 cycles of 95°C for 20 s, 55°C to 64°C (primer annealing temperature) for 15 s, and elongation at 72°C for 10 s. Subsequent melt curve analysis was performed by heating to 95°C for 10 s, cooling to 65°C for 1 min, and heating to 97°C. Primer sequences and accession numbers are outlined in Table 2. Relative expression levels of all genes were calculated relative to the housekeeping gene β-actin using LightCycler 480 software (Roche Diagnostics GmbH, Mannheim, Germany). Data represent means from 6 biological replicates in duplicates.

**TABLE 2:** Primers used for real-time PCR^*a*^

Gene	GenBank accession no.	Direction	Primer sequence	*T_m_* (°C)	Product size (bp)
LAMP-1	NM_205283.2	FWD	CTTGCCGGTCTGGTTCTGAT	60	166
		REV	GGGGAGAGATAGGGGTGGTT		

SQLE	NM_001194927.1	FWD	CGCTGACGGTTGTAGCTGAT	60	200
		REV	AAGGACGCGAGTCTCAGTTG		

FDFT1	NM_001039294.1	FWD	CTACCCTCTGCTGCAAGGTC	60	195
		REV	TTAGAAACGTGGCCCACTCG		

HMG-CoA reductase	AB109635.1	FWD	GCTATGGCTGGTAGCATAGGT	60	145
		REV	TCACTGGTGGAACAGTACGCT		

Cytoplasmic beta actin	X00182	FWD	TGCTGTGTTCCCATCTATCG	60	150
		REV	TTGGTGACAATACCGTGTTCA		

a FDFT1, farnesyl-diphosphate farnesyltransferase 1; FWD, forward; REV, reverse.

### Western blotting.

Samples were lysed in lysis buffer in the presence of protease inhibitors (Thermo Fisher Scientific, Paisley, UK). The lysates were suspended in sample loading buffer (Sigma-Aldrich/Merck, USA) and loaded in a 10% SDS-PAGE gel. After semidry transfer of SDS-PAGE gels, nitrocellulose membranes were blocked (5% skimmed milk powder in PBS for 2 h at RT). Membranes were incubated overnight (4°C) with primary antibodies, rabbit monoclonal antibody to chicken tubulin, rabbit monoclonal antibody to SqE, and rabbit monoclonal antibody to HMG-CoA, in the respective blots. The next day, membranes were washed (0.5% skimmed milk powder in PBS) and incubated (5% skimmed milk powder in PBS for 2 h at RT) with a secondary antibody, donkey pAb to goat IgG Red, in the respective blots. Finally, blots were probed with the Odyssey CLx imaging system (Li-Cor, USA), bands were quantified with Image Studio Lite software, and images were processed using Adobe Photoshop software.

### Flow cytometry.

The viabilities of CEFs treated with the pharmacological inhibitors or diluent (vehicles) were determined to identify their nontoxic concentrations. Each pharmacological inhibitor was titrated, and CEFs were exposed to the inhibitors for 72 h.

### Viability assay.

Trypsinized CEFs were stained with 7-AAD (BD Biosciences, Oxford, UK) and acquired using a MACSQuant flow cytometer, and cell viability was analyzed using FlowJo software. Nontoxic concentrations of the inhibitors and chemicals were selected based on cell viability determined using flow cytometry data and confluence. CEFs were acquired in triplicates from 4 independent experiments using a MACSQuant flow cytometer, and the results were analyzed using FlowJo software.

### Fluorescence confocal microscopy.

Seventy-two hours after mock infection or infection with the RB1B virus in the presence/absence of U18666A or shRNA and pScrambled-overexpressing CEFs targeting LAMP-1, the samples were prepared for imaging. CEFs were trypsinized and seeded in 24-well plates that contained 12-mm-diameter round coverslips at a rate of 1.0 × 10^5^ cells per well. In brief, mock-infected or infected CEFs were fixed with 4% formaldehyde for 30 min at RT and washed twice with PBS. Cells were subsequently permeabilized (where indicated) with 0.1% Triton X-100 buffer solution, blocked (1 h in 0.5% BSA–PBS), and incubated overnight (4°C) with anti-gB (mouse IgG2b; 1 μg/500 μl in 0.5% BSA–PBS) or anti-LAMP-1 (mouse IgG1; 1 μg/ml in 0.5% BSA–PBS). Cells were washed twice with PBS and again incubated overnight (4°C) with anti-IgG2b-568 nm (goat anti-mouse, 0.5 μg/500 μl in 0.5% BSA–PBS) or goat anti-IgG1-568 nm (goat anti-mouse, 0.5 μg/500 μl in 0.5% BSA–PBS). The next day, nuclei were labeled with DAPI. Coverslips were mounted in Vectashield mounting medium for fluorescence imaging.

### (i) Cholesterol staining.

Cellular localization of cholesterol based on filipin III staining was performed according to the manufacturer’s recommendations (Abcam Ltd., Cambridge, UK). In brief, CEFs were fixed in the cell-based assay fixative solution for 10 min (RT in the dark). Cells were subsequently washed and stained (1 h at RT in the dark) with filipin III (1:100 dilution of a filipin III stock solution in cholesterol detection assay buffer). After a final wash, coverslips were mounted in Vectashield mounting medium for fluorescence imaging (excitation of 340 to 380 nm and emission of 385 to 470 nm).

### (ii) TopFluor cholesterol staining.

CEF cells were either mock infected or infected with RB1B virus in maintenance medium containing 628 nM TopFluor cholesterol for 72 h, and samples were prepared for confocal imaging.

### (iii) Lipid droplet staining.

CEF cells were either mock infected or infected with RB1B virus in maintenance medium containing 628 nM cholesterol for 72 h, and samples were prepared for confocal microscopy. TopFluor cholesterol and lipid droplet groups were stained with Alexa Fluor 568 goat anti-mouse IgG2b and Alexa Fluor 488 goat anti-mouse IgG2b (Thermo Fisher Scientific, Paisley, UK) secondary antibodies, respectively. Further lipid droplets groups were stained with HCS LipidTOX red neutral lipid stain.

### (iv) Visualization.

Cells were viewed using a Leica SP2 laser scanning confocal microscope, and optical sections were recorded using either 663 or 640 nM with a numerical aperture of 1.4 or 1.25, respectively. All data were collected sequentially to minimize cross talk between fluorescence signals. The data are presented as maximum projections of z-stacks (23 to 25 sections; spacing of 0.3 mm). Maximum projections of z-stacks were analyzed with LAS AF Lite software for localization of the relative fluorescence intensity across a straight line.

### (v) Manders’ colocalization coefficient.

All images were processed using Fiji software, and Manders’ colocalization coefficient was calculated using the colocalization (COLOC) function for a specified region of interest (ROI). Manders’ M1 and M2 weighted coefficients were calculated to determine the extent of colocalization between a pair of fluorescent signals and imaged in two channels. Manders’ M1 determines the degree of channel colocalization (gB with filipin II, gB with LAMP-1, and LAMP-1 with filipin III), and M2 determines the reverse. The uptake of fluorescent cholesterol by mock- and RB1B-infected CEFs was analyzed using ImageJ and estimated as the mean fluorescence intensity (MFI) per cell. For estimation of lipid droplet formation, in total, 15 z-stack cells from mock- and RB1B-infected cells were analyzed using IMARIS (Bitplane Scientific Software).

### Statistical analysis.

All data are presented as means ± standard deviations (SD) from at least three independent experiments. Quantification was performed using GraphPad Prism 7 for Windows. The differences between groups, in each experiment, were analyzed by one-way analysis of variance (ANOVA) followed by a Tukey multiple-comparison test to identify those groups that differed if the ANOVA result was significant. Results were considered statistically significant at a *P* value of *<*0.05 (*).
